# Elicitation of Neutralizing Antibodies Directed against CD4-Induced Epitope(s) Using a CD4 Mimetic Cross-Linked to a HIV-1 Envelope Glycoprotein

**DOI:** 10.1371/journal.pone.0030233

**Published:** 2012-01-24

**Authors:** Antu K. Dey, Brian Burke, Yide Sun, Klara Sirokman, Avishek Nandi, Karin Hartog, Ying Lian, Anthony R. Geonnotti, David Montefiori, Michael Franti, Grégoire Martin, Andrea Carfi, Pascal Kessler, Loïc Martin, Indresh K. Srivastava, Susan W. Barnett

**Affiliations:** 1 Novartis Vaccines and Diagnostics, Cambridge, Massachusetts, United States of America; 2 Department of Surgery, Duke University Medical Center, Durham, North Carolina, United States of America; 3 CEA, iBiTecS, Service d'Ingénierie Moléculaire des Protéines, Gif sur Yvette, France; The University of Hong Kong, Hong Kong

## Abstract

The identification of HIV-1 envelope glycoprotein (Env) structures that can generate broadly neutralizing antibodies (BNAbs) is pivotal to the development of a successful vaccine against HIV-1 aimed at eliciting effective humoral immune responses. To that end, the production of novel Env structure(s) that might induce BNAbs by presentation of conserved epitopes, which are otherwise occluded, is critical. Here, we focus on a structure that stabilizes Env in a conformation representative of its primary (CD4) receptor-bound state, thereby exposing highly conserved “CD4 induced” (CD4i) epitope(s) known to be important for co-receptor binding and subsequent virus infection. A CD4-mimetic miniprotein, miniCD4 (M64U1-SH), was produced and covalently complexed to recombinant, trimeric gp140 envelope glycoprotein (gp140) using site-specific disulfide linkages. The resulting gp140-miniCD4 (gp140-S-S-M64U1) complex was recognized by CD4i antibodies and the HIV-1 co-receptor, CCR5. The gp140-miniCD4 complex elicited the highest titers of CD4i binding antibodies as well as enhanced neutralizing antibodies against Tier 1 viruses as compared to gp140 protein alone following immunization of rabbits. Neutralization against HIV-2_7312/V434M_ and additional serum mapping confirm the specific elicitation of antibodies directed to the CD4i epitope(s). These results demonstrate the utility of structure-based approach in improving immunogenic response against specific region, such as the CD4i epitope(s) here, and its potential role in vaccine application.

## Introduction

The human immunodeficiency virus type 1 (HIV-1) envelope glycoprotein (Env) is the lone viral gene product exposed on surface of the virus and therefore is the major target of HIV-1-specific neutralizing antibodies (NAbs). This trimeric glycoprotein mediates receptor binding and viral entry through its interactions with both CD4 and CCR5/CXCR4. These characteristics make Env a logical candidate as a component of an HIV-1 vaccine. Such a vaccine will likely need to elicit broadly cross-neutralizing antibodies to be effective. However, due to extensive intra- and inter-subtype sequence variability of Env, anti-Env antibody response are generally isolate-specific. To be effective against diverse isolates, the NAbs induced by a prospective vaccine will need to recognize multiple Env variants, as patients are unlikely to encounter viruses that match the vaccine strain during natural infection. Therefore, the ideal method of generating a broad, cross-reactive NAb response would be to target highly conserved regions in Env [1]. Unfortunately, many conserved epitopes of Env are occluded or transiently exposed, and therefore are poorly immunogenic. Several monoclonal antibodies (MAbs) have been isolated from patients with broad, potent-neutralizing activity [2], [3], [4], [5], [6], [7], however attempts to elicit similarly potent and broadly NAbs by vaccination using recombinant forms of Env as antigens have, at best, met limited success [8], [9]. The antibodies generated in this manner are primarily strain-specific and have limited breadth in their neutralizing activity. Designing immunogens that are able to elicit antibodies that are broadly cross-neutralizing is a challenge as accessible immunodominant variable regions redirect antibody responses away from the desired, but often masked conserved regions [1], [10], [11], [12], [13]. Thus, many studies involving Env have focused on attempting to dampen the immunogenicity of the highly variable regions and/or to increase the immunogenicity of the desired conserved epitopes [8], [14], [15], [16].

One such conserved region that has been under evaluation is the conformational epitope that Env forms upon binding with the receptor CD4. This CD4-induced (CD4i) epitope is highly conserved [10]. In fact, many CD4i antibodies are quite potent, even against the highly divergent HIV-2, in the presence of sCD4, indicating the conserved nature of this epitope [10]. This make the CD4i epitope(s) an attractive target for vaccine development. A potential concern that CD4i antibodies, as whole IgG, face steric hindrance in accessing the epitope [17] and hence not effective in neutralizing primary isolates remain. However, broadly cross-reactive neutralizing antibodies were elicited in rhesus macaques by using covalently cross-linked HIV-1 Env-CD4 receptor complexes [18]. DeVico et al. also showed in a SHIV-challenged model that antibodies to CD4i sites in HIV-1 gp120 correlated with the control of SHIV challenge in macaques vaccinated with cross-linked and single-chain gp120–CD4 complexes [19]. Importantly, it was shown recently that humans infected with HIV-1 generate CD4i antibodies during the course of infection [20], [21], [22], [23]. Additionally, it was observed that there is a correlation between the presence of CD4i NAbs and protection from disease [19], [22]. Also in human clinical trials focusing on induction of Env-specific antibodies, either using protein alone, viral vector prime-protein boost or DNA prime-protein boost, analysis of serum antibody response show elicitation of neutralizing antibodies to CD4i sites [24], [25]. Therefore, in an effort to create improved Env antigens that would effectively display cryptic CD4i epitope(s) without using the native CD4 molecule, which could present safety concerns in a vaccine preparation due to potential autoimmune reactions, studies using peptide mimetics of CD4 were performed [26], [27], [28], [29]. These were based on the scyllatoxin-scaffold and several generations were evaluated to define the optimal molecule for use in complex with HIV-1 Env [26], [27], [28], [29]. Using the most advanced of these CD4-mimetic miniprotein (miniCD4), M64U1-SH, to generate stable site-directed disulfide-linked complexes with SF162 gp120 or oligomeric V2 loop-deleted gp140 (gp140ΔV2), the MAb and CCR5 binding profiles were found to be analogous to those obtained using the same Env antigens bound to soluble CD4 [27]. In the present study, the immunogenicity of a stably cross-linked gp140-miniCD4 (gp140-S-S-M64U1) complex was evaluated to determine the merit of this approach for the elicitation of antibodies directed against highly conserved CD4i epitope(s) and virus neutralization.

## Materials and Methods

### Ethics Statement

The study was fully approved (approval no. 09 NVD 044.3.3.09) by the Institutional Animal Care and Use Committee at Novartis in accordance with the requirements for the humane care and use of animals as set forth in the Animal Welfare Act, the Institute for Laboratory Animal Research (ILAR) Guide for the care and use of laboratory animals, and all applicable local, state and federal laws and regulations.

### Reagents

Monoclonal antibodies (MAbs), b12, 2G12, 2F5 and 4E10 were purchased from Polymun Scientific (Vienna, Austria). MAb 17b was kindly provided by Dr. James Robinson. The biotinylated tyrosine-sulfated (at positions 10 and 14) N-terminal 22-mer CCR5 peptide [30], [31] and control biotinylated, non-sulfated, N-terminal 22-mer CCR5 peptide were purchased from American Peptide Co. (Sunnyvale, CA). Soluble CD4 (sCD4) was purchased from Progenics Pharmaceuticals (Tarrytown, NY).

### Generation and characterization of Env proteins

Two forms of recombinant HIV-1 Envs were used in this study: gp140 (and its cross-linking or mixing with miniCD4 to form the complex) as immunogens and gp120 for *in vitro* serum mapping analysis. Both recombinant Envs, gp140 and gp120, were derived from subtype B CCR5-tropic strain HIV-1_SF162_. The gp120 glycoprotein is discussed in later section. The oligomeric gp140 glycoprotein contained a 30 amino acid deletion in the V2 loop region, as described previously [16], [32], and were produced in stable CHO-cell lines [32]. The gp140 glycoprotein was purified using a three-step purification process involving *Galanthus Nivalis*-Agarose (GNA) affinity column, cation exchange DEAE column and a final ceramic hydroxyapatite (CHAP) column as described by Srivastava et al. [33]. The purified gp140 glycoprotein was then analyzed by SDS-PAGE for level of purity and immunoblot for specific reactivity (to anti-SF162 gp140 polyclonal rabbit sera). The purity and homogeneity of purified gp140 glycoprotein, as determined by SDS-PAGE, was >98%. Using Surface Plasmon Resonance (SPR) (BIAcore 3000), the binding of gp140 glycoprotein to sCD4, MAbs b12 and 2G12, were analyzed, as previously described [34].

For serum mapping, various gp120 glycoproteins were also generated and characterized. Using transient transfection of HEK293T cell lines, the following gp120 glycoproteins were produced: gp120wt (wild-type), gp120ΔV1V2 (V1V2 deleted), gp120ΔV3 (V3 deleted), gp120D368R (CD4-binding site mutant) and gp120I420R (CD4i-binding site mutant). 48 h post-transfection, supernatants were collected and clarified off cellular debris via high-speed centrifugation and concentrated approximately 10-fold using 100 kDa-cut off membrane filter. The concentrated materials were stored at −80°C in presence of 1 mM EDTA, 1 mM EGTA and complete protease inhibitor cocktail (Boehringer-Mannheim). The concentrated supernatants were later thawed at 4°C and purified, as described above. The purified gp120 glycoproteins were then analyzed by SDS-PAGE for level of purity and immunoblot for specific reactivity (to anti-SF162 gp140 polyclonal rabbit sera). Both data showed that there was no major aggregation, degradation or proteolysis and that the purified gp120 glycoproteins were homogeneous and the level of purity was >98%.

All the gp120 glycoproteins, wild-type and mutants, were then subjected to SPR binding analysis for characterizing their antigenic profiles. All assays were performed at 25°C using HBS-EP buffer (10 mM HEPES, pH 7.4, 150 mM NaCl, 3 mM EDTA, 0.005% [v/v] Surfactant P20; BIAcore, Uppsala, Sweden), which was degassed for 1 h before use. Approximately 3000 RUs of CD4-IgG2 and MAbs (b12 – CD4BS; 2G12 – glycan-specific; 3019a, 2557 – anti-V3 and 17b – CD4i) were immobilized via amine coupling on a CM5 sensor chip using manufacturer's protocol, as described elsewhere [34]. 100 nM of gp120, wild-type or mutant, were injected at 10 µl/minute and binding to various ligands analyzed. Following each run, the sensor surface was regenerated using two 10 µl injections of 5 mM and 10 mM NaOH. The data were analyzed using BIAevaluation software 3.2 (BIAcore Inc). To correct for refractive index changes and instrument noise, the response data from the control mock-treated flow-cell were subtracted from those obtained from the experimental flow-cell.

### Generation and characterization of miniCD4 and gp140-miniCD4 complex

The CD4-mimetic miniprotein (miniCD4), M64U1 [28] and M64U1-SH [27], were synthesized by solid phase methods as described previously [26], [27], [29], [35]. Cross-linking of gp140-S-S-M64U1 was performed in the following manner: M64U1-SH, having an additional sulfhydryl group on side chain of Lys4, was incubated at a molar ratio of 3∶1 with SF162 gp140 for 1 h at room temperature (RT). The additional SH group was introduced derivatizing the position 4 of the M64U1 peptide [28].

To characterize the gp140-S-S-M64U1 complex for its ability to bind various MAbs, Surface Plasmon Resonance (SPR) (BIAcore 3000) was used. All assays were performed at 25°C using HBS-EP buffer (10 mM HEPES, pH 7.4, 150 mM NaCl, 3 mM EDTA, 0.005% [v/v] Surfactant P20; BIAcore, Uppsala, Sweden), which was degassed for 1 h before use. The flow-rate was maintained at 10 µl/minute. A CM5 sensor chip was used and ≤1500 Response Units (RUs) of sCD4 (Progenics Pharmaceuticals, Tarrytown, NY) or ∼3000 RUs of MAbs (b12, 2G12, 17b) were covalently immobilized via amine coupling using manufacturer's protocol, as described elsewhere [34]. For the CCR5 peptide-binding analysis, 800–900 RUs of biotinylated sulfated N-terminal 22-mer CCR5 peptide, [Biotin-CCR5 (SO3)] [30], [31], and the control, biotinylated non-sulfated N-terminal 22-mer CCR5 peptide [Biotin-CCR5 (control)] were immobilized on a streptavidin (SA) sensor chip; the free streptavidin surface was blocked by biotin. Purified SF162 gp140 (100 nM) or cross-linked gp140-S-S-M64U1 complex (100 nM) was then injected at 10 µl/minute for binding analysis. Following each run, the sensor surface was regenerated using two 10 µl injections of 5 mM and 10 mM NaOH. The data were analyzed using BIAevaluation software 3.2 (BIAcore Inc). To correct for refractive index changes and instrument noise, the response data from the control mock-treated flow-cell were subtracted from those obtained from the experimental flow-cell.

### Rabbit Immunization

Immunization studies were conducted at Josman LLC (Napa, CA), a research facility that is licensed through the USDA (No. 93-R-0260) and has a Public Health Service (PHS) Assurance from the NIH (No. A3404-01). Five female New Zealand White rabbits, per group, were used in the immunogenicity study. Rabbits were immunized with M64U1-SH cross-linked with gp140 (gp140-S-S-M64U1), gp140 mixed with M64U1 (mixed, no cross-linking), gp140 and M64U1 (miniCD4, as control). Four protein immunizations were administered intramuscularly, in the gluteus, at weeks 0, 4, 12, and 24. The total protein dosage at each immunization was 50 µg. Proteins were administered in MF59, Novartis's proprietary oil-in-water adjuvant. Serum samples were collected prior to first immunization and two weeks following each immunization. The study was fully approved (Approval no. 09 NVD 044.3.3.09) by the Institutional Animal Care and Use Committee at Novartis in accordance with the requirements for the humane care and use of animals as set forth in the Animal Welfare Act and the Institute for Laboratory Animal Research (ILAR) Guide for the care and use of laboratory animals, and all applicable local, state and federal laws and regulations.

### HIV-1 Env pseudovirus neutralization assays

Virus neutralization titers were measured using a well-standardized assay employing HIV-1 Env pseudoviruses and a luciferase reporter gene assay in TZM-bl cells [Dr. John C. Kappes, Dr. Xiaoyun Wu and Tranzyme, Inc. (Durham, NC)] as described previously [36], [37]. Briefly, a total of 200 TCID50 pseudoviruses/well were added to diluted serum samples and incubated at 37°C for 1 h. Following incubation, 10,000 cells/well in DEAE-dextran-containing media were added and incubated for 48 h at 37°C. The final concentration of DEAE-dextran was 10 µg/ml. After a 48 h incubation, 100 µl of cells was transferred to a 96-well black solid plates (Costar®) for measurements of luminescence using Bright Glo substrate solution as described by the supplier (Promega). Neutralization titers are the dilution at which relative luminescence units (RLU) were reduced by 50% compared to virus control wells after subtraction of background RLUs. HIV-1 Env pseudoviruses were prepared by co-transfection of HEK293T cells with expression plasmids containing full-length molecularly cloned gp160 *env* genes from a panel of HIV-1 isolates combined with an *env*-deficient HIV-1 backbone vector (pSG3Δenv) using FuGENE-6 HD (Roche Applied Sciences, Indianapolis, IN), as previously reported [36]. After 48 h, the cell culture supernatants containing the pseudoviruses were filtered through a 0.45 µm filters and stored at −80°C until use.

### HIV-2 neutralization assays

For the detection of CD4i neutralizing antibodies, a modified neutralization assay was used, as previously described [23].

### Antibody avidity measurements

Antibody avidity index determination was performed using an ammonium thiocyanate (NH_4_SCN) displacement ELISA, as described elsewhere [33].

### Measuring envelope-specific antibody titers by ELISA

Envelope-specific total antibody titers in sera immunized with gp140 or cross-linked gp140-miniCD4 (gp140-S-S-M64U1) complex were quantified by a standard ELISA assay, as previously described [33].

### Serum mapping - Generation of antigen-coupled beads

To dissect specificity of antibody response against epitope(s) on gp140 or the gp140-miniCD4 (cross-linked or mixed) complex, a ‘serum mapping’ assay as described by Y Li et al. [38] was used. Following the expression, purification and characterization of the gp120 variants, the proteins including cross-linked gp140-S-S-M64U1 complex, were coupled to paramagnetic polystyrene, tosylactivated magnet MyOne Dynabeads (Invitrogen). Coupling was performed at 37°C for 6–8 h according to the manufacturer's instructions, briefly described as follows. 1 mg of protein, which has been extensively dialyzed against PBS (pH 7.4), was coupled to 50 mg (0.5 ml volume) of paramagnetic beads, in 1.25 ml of coupling buffer [0.1 M sodium borate buffer (pH 9.5) with 1 M ammonium sulfate]. The antigen-coupled beads were separated from the coupling buffer with a magnet. The antigen-coupled beads were then resuspended with 5 ml of blocking buffer [PBS (pH 7.4) with 0.5% (w/v) bovine serum albumin (BSA) and 0.05% (v/v) Tween 20] and incubated at 4°C for 14–16 h. The blocking buffer was removed by aspiration, and the antigen-coupled beads were washed extensively with 5 ml of buffer [PBS (pH 7.4) with 0.1% (w/v) BSA and 0.05% (v/v) Tween 20]. Finally, the beads were resuspended in 0.5 ml of storage buffer [PBS (pH 7.4) with 0.1% (w/v) BSA, 0.05% (v/v) Tween 20, 0.02% (v/v) sodium azide, and protease inhibitors] and stored at 4°C until further use.

After the generation of antigen-coupled beads, the antigenic integrity and specificity of each protein were verified by adsorption of ligands followed by D7324-capture [anti-gp120 C5 region polyclonal antibodies (Aalto, Dublin, Ireland) at 5 µg/ml] ELISA. The ligands used during adsorption to validate the antigen-coupled beads were CD4-IgG2 (against D368R gp120) and MAbs b12 (against D368R gp120), 2G12 (as control against all gp120s), 17b (I420R gp120 & gp140-S-S-M64U1 complex) and 3019a, 2557 (ΔV3 gp120). All antigens, including gp140-S-S-M64U1 complex, upon coupling to paramagnetic beads maintained their characteristic binding profiles and their ability to deplete specific antibody, as observed by ELISA (data not shown). Therefore, the beads were qualified to be used in subsequent ‘serum mapping’ analysis. BSA-coated beads were also generated using the same approach, as described above, to be used as negative control for the solid phase adsorption process.

### Serum mapping - Depletion and antibody-binding ELISA

Before adsorption, the gp120 antigen-coupled beads (including BSA-coated beads, negative control) were washed 2–3 times with Dulbecco modified Eagle medium (DMEM) containing 10% (v/v) fetal bovine serum (FBS) and incubated in the same media at room temperature for 20–30 min to block potential nonspecific binding to the beads prior to adsorption. Rabbit sera were then diluted 10-fold in DMEM containing 10% (v/v) FBS; 500 µl of the diluted sera was incubated with 50 µl of beads at room temperature for 1 h. Following adsorption, the beads were removed from the treated serum samples with a magnet. The ‘depleted’ serum were collected and stored at 4°C until subsequent analysis by ELISA. The antigen-coupled beads were washed twice with PBS (pH 7.4) containing 500 mM NaCl and once with PBS (pH 7.4). The bound antibodies were then eluted by a stepwise decrease in pH – first using 125 µl 100 mM glycine-HCl elution buffer (pH 2.7) for 30 s, separation of the beads with a magnet and then subjecting the acid-eluted solution containing IgG to a separate tube containing 1 M Tris (pH 9.0) buffer for neutralization. Subsequently, the same procedure was performed using 125 µl 100 mM glycine-HCl (pH 2.2) to recover any IgG resistant to elution at pH 2.7. This sample is referred to as ‘adsorbed’ sample.

The presence of gp120-specific antibodies in the adsorbed and depleted fractions were analyzed by Ab D7324-capture ELISA, using a protocol described previously [39], [40]. The secondary antibody used was ECL™ Donkey HRP-linked anti-rabbit IgG (GE Healthcare) and used at 1∶10,000 dilutions (100 µl/well). The optical density (OD) was determined using a microplate reader (Molecular Devices) at 450 nm.

### V3-peptide competition assay

The V3-peptide used in this competition assay was SF162 V3-cyclic peptide (CTRPNNNTRKSITIGPGRAFYATGDIIGDIR QAHC) [41]; a V3-scrambled cyclic peptide (CTRPNNNTRKSIFYRGAPGITAT GDIIGDIRQAHC) was used as control. The competition assay was performed as described below. Briefly, maxisorb (Nunc™) 96-well plates were coated with 100 µl of 2 µg/ml (200 ng/well) of Ab D7324 [anti-gp120 C5 region polyclonal antibodies (Aalto, Dublin, Ireland)] in PBS (pH 7.4) and incubated overnight at 4°C. The plates were then blocked with 200 µl of 1% (w/v) BSA in PBS (pH 7.4) with incubation at 37°C for 1 h. The plates were washed three times with wash buffer [PBS (pH 7.4)+0.1% (v/v) Tween 20]. SF162 gp140 protein was diluted at 1 µg/ml in dilution buffer [PBS (pH 7.4)+1% (w/v) BSA+0.1% (v/v) Tween 20] and 100 µl of 1 µg/ml of protein added (100 ng/well) onto the Ab D7324 coated-plate and incubated at room temperature for 1 h. The plates were then washed three times with wash buffer. The rabbit sera were incubated with either V3-cyclic peptide or V3-scrambled cyclic peptide (control), for 1 h at room temperature, with varying concentrations of V3-peptide (serial dilutions in dilution buffer) before their addition to the gp140-captured ELISA plates. The peptide+sera dilutions were then incubated at room temperature for 2 h. The plates were then washed three times with wash buffer and bound anti-Env rabbit antibodies detected with ECL™ Donkey HRP-linked anti-rabbit IgG (GE Healthcare) at 1∶10,000 dilutions (100 µl/well) followed by development of the reaction using TMB Microwell Peroxidase Substrate System (KPL). The percent (%) inhibition, which is the extent of serum binding to gp140 inhibited by V3-peptide, is calculated as the ratio of serum binding in presence of V3-cyclic peptide to serum binding in presence of control peptide ×100.

### Statistical analyses

Comparisons between multiple groups were carried out using an analysis of variance (ANOVA). A two-sided Wilcoxon rank sum analysis was used to test for differences between immunization groups. For all comparisons, a two-sided P<0.05 was considered statistically significant. All analyses were performed using the analysis software within the GraphPad Prism package 5.01.

## Results

Based on a preliminary study using the CD4-mimetic miniprotein (miniCD4), M64U1-SH, coupled to soluble HIV-1 gp120 [27] and gp140ΔV2 glycoproteins, the cross-linked gp140-S-S-M64U1 complex was chosen for this study. Since, the SF162 V2-loop deleted gp140 (gp140ΔV2) immunogen was previously shown to elicit higher titers of cross-reactive neutralizing antibodies than SF162 gp140 [32], the modified V2 loop-deleted gp140 (gp140ΔV2), hereafter referred to as oligomeric gp140 (gp140), was used for these studies.

### The stably cross-linked gp140-miniCD4 (gp140-S-S-M64U1) complex showed enhanced exposure of CD4i epitope(s) *in vitro*

**Figure 1:**
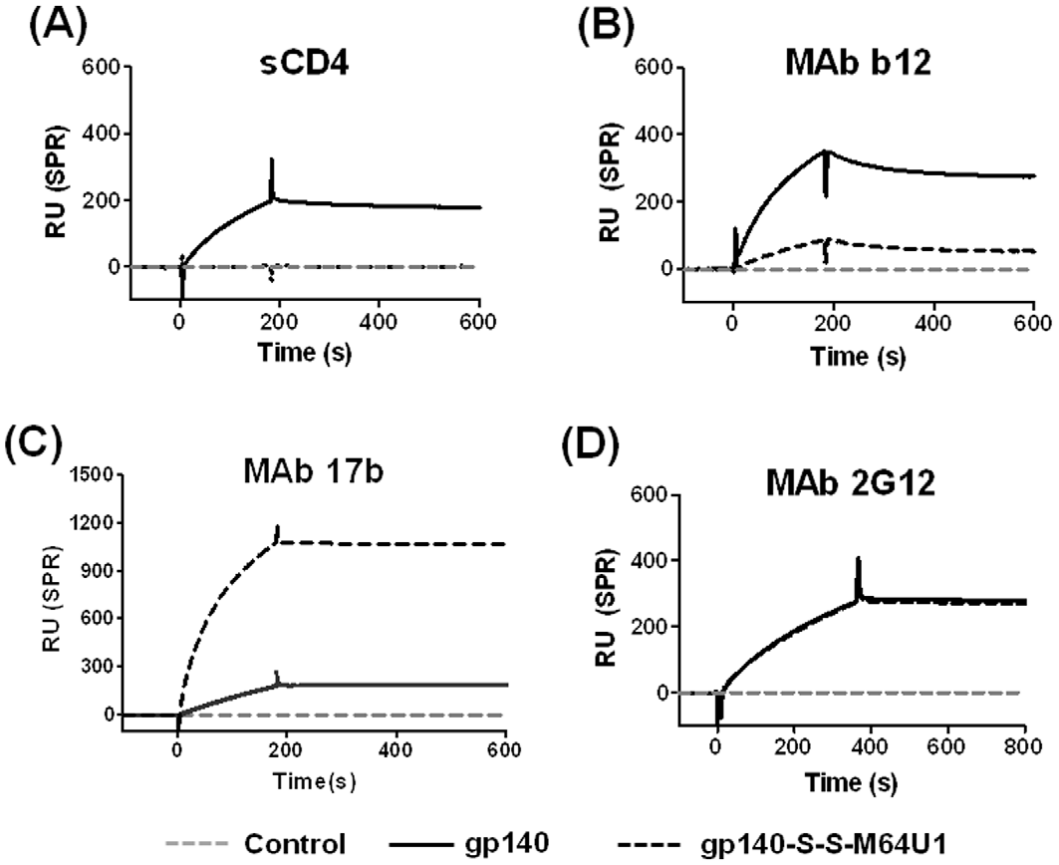
Binding of cross-linked gp140-miniCD4 (gp140-S-S-M64U1) complex to soluble CD4 and MAbs. Surface Plasmon Resonance (SPR) binding analysis of cross-linked gp140-miniCD4 (gp140-S-S-M64U1) complex, in comparison to uncomplexed gp140, to ligands immobilized on CM5 sensor chip: (A) soluble CD4 (sCD4), (B) (CD4 binding site), (C) MAb 2G12 (glycans), and (D) MAb 17b (CD4i site).

After generation of cross-linked gp140-miniCD4 (gp140-S-S-M64U1) complex [27], the antigenicity and stability of the complex was analyzed and compared to that of uncomplexed gp140 glycoprotein by Surface Plasmon Resonance (SPR) to measure binding of the complex (and gp140) to covalently immobilized soluble CD4 (sCD4) or MAbs (b12, 2G12, 17b). While gp140 bound sCD4, the gp140-S-S-M64U1 complex in which the CD4BS cavity was occupied by the miniCD4, did not (Fig. 1A). Similarly, while uncomplexed gp140 bound to MAb b12 with high affinity as expected, the binding of gp140-S-S-M64U1 complex to MAb b12 was reduced by >4-fold, again due to the miniCD4 occupying the CD4BS of the gp140 (Fig. 1B). Most importantly, while uncomplexed gp140 bound to the CD4i epitope specific MAb, 17b, poorly, reflecting relatively little exposure of this CD4i epitope on the non-receptor bound gp140; in contrast, the gp140-S-S-M64U1 complex was efficiently recognized by MAb 17b. The binding of MAb 17b was increased by >5-fold (Fig. 1C), indicative of the enhanced exposure of the CD4i epitope(s) in the complex. These data validated the antigenic state of the complex. Of note, MAb 2G12, a MAb that recognizes terminal mannose on HIV-1 Env, bound both the complex and uncomplexed gp140s identically (Fig. 1D).

**Figure 2:**
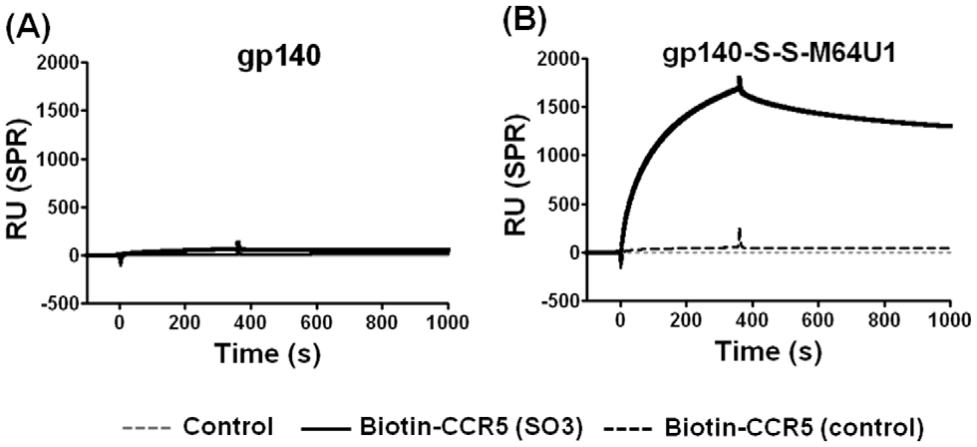
Binding of cross-linked gp140-miniCD4 (gp140-S-S-M64U1) complex to CCR5 peptide. Surface Plasmon Resonance (SPR) binding analysis of (A) gp140 and (B) cross-linked gp140-miniCD4 (gp140-S-S-M64U1) complex to two ligands immobilized on CM5 sensor chip: biotinylated, tyrosine-sulfated (at positions 10 and 14) 22-mer N-terminal CCR5 peptide [biotin-CCR5(SO3)] [30], [31] and a control, biotinylated non-sulfated 22-mer N-terminal CCR5 peptide [biotin-CCR5 (control)].

The binding of gp140-S-S-M64U1 complex to tyrosine-sulfated CCR5 N-terminal 22-mer peptide [31] was also measured using an approach described previously [30]. While the gp140-S-S-M64U1 complex recognized the sulfated CCR5-peptide [biotin-CCR5 (SO3)] efficiently and specifically (as opposed to non-sulfated CCR5 peptide, used as control), the gp140 in its native state did not (Fig. 2A, B). This analysis further support that the gp140-S-S-M64U1 complex was in a CD4-induced state and was thus likely a good immunogen with the potential to elicit antibodies directed toward CD4i epitope(s) on HIV-1 Env.

### Elicitation of high titer antigen-specific antibody responses using gp140 and gp140-miniCD4 complexes

**Figure 3:**
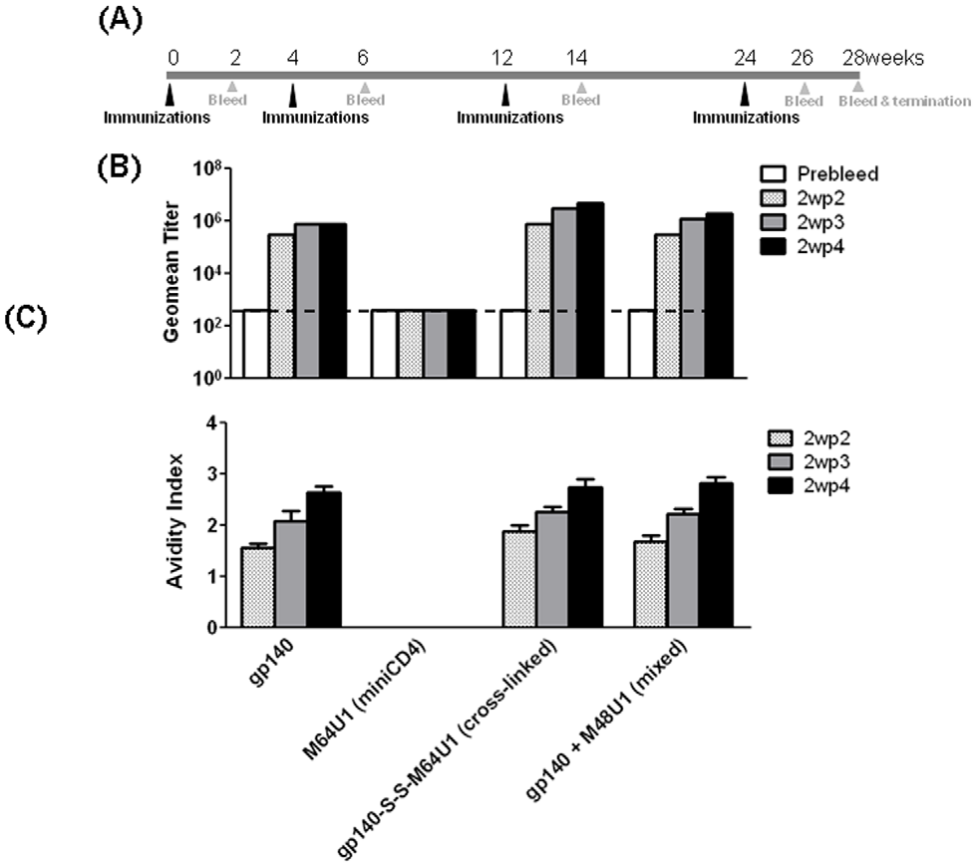
Rabbit study design and antibody binding analysis of vaccine sera. (A) Scheme of vaccination study in rabbits, showing time-point of administration of immunogens and collection of sera for evaluation. (B & C) SF162 gp140-specific binding antibody responses induced by various immunogens [gp140, M64U1 (miniCD4), gp140-S-S-M64U1 (cross-linked), or gp140+M64U1 (mixed)] in rabbit sera. Two weeks following the second (2wp2), third (2wp3) and fourth (2wp4) immunizations, individual sera were analyzed via ELISA and group geometric mean (geomean) titers calculated. Pre-immune sera were also evaluated to identify the level of non-specific reactivity. (B) Avidity of SF162 gp140-specific antibodies from the four groups of animals at 2wp2, 2wp3 and 2wp4 following immunization.

An immunization study was performed in rabbits to test whether or not the cross-linked gp140-miniCD4 (gp140-S-S-M64U1) complex could elicit Ab responses directed to the CD4i epitope(s) on the HIV-1Env in an animal model where there would be an absence of the cognate CD4 receptor ligand for the HIV-1 Env protein. This type of study would serve as the strictest test of whether the CD4i sites were being exposed *in vivo* as a result of complexing of the gp140 to the CD4-mimetic miniprotein (miniCD4) as opposed to the natural receptor molecule as might be expected and has been observed in non-human primates [17](W Bogers, SW Barnett, et al., unpublished results). The scheme of the rabbit immunization is shown in Fig. 3A. Four protein immunizations were administered using MF59 adjuvant at 0, 4, 12, and 24 weeks and sera were collected post-immunization at intervals, as indicated in Fig. 3A.

To determine the level of gp140-specific antibodies that were generated by immunization, serum samples were collected before immunization (pre-immune sera) and 2-weeks following each immunization, including 4-weeks after last immunization, and analyzed by ELISA. Sera from all immunization groups elicited antibodies that recognized gp140 above the background pre-immunization levels following the initial immunization (Fig. 3B). Binding titers were increased with additional immunizations and achieved the highest titers two weeks following the third (2wp3) or fourth (2wp4) immunization for all groups, except M64U1 (miniCD4) group that was used as a control. In animals immunized with M64U1 (miniCD4), gp140-specific antibody titers did not increase beyond the basal level (Fig. 3B, shown by the dotted line) observed for the pre-immune sera.

Most noteworthy, when the total anti-gp140 binding antibodies were analyzed by ELISA, a ∼10-fold increase was observed between the titers generated by cross-linked gp140-S-S-M64U1 complex in comparison to gp140 alone. At two-weeks post-fourth immunization (2wp4), while gp140 elicited a binding antibody titer of ∼10^6^, the cross-linked gp140-S-S-M64U1 complex generated titers about a log higher (∼10^7^) reaching statistical significance [for 2wp4 sera, gp140 vs. gp140-S-S-M64U1 (cross-linked), p = 0.0079; gp140 vs. gp140+M64U1 (mixed), p = 0.0556]. The gp140+M64U1 (mixed) complex on the other hand elicited antibody titers (≥10^6^) to a level that was not statistically different from those elicited by gp140 alone, but were lower than those generated by the cross-linked gp140-S-S-M64U1 complex at 2wp4 (Fig. 3B). As expected, M64U1 (miniCD4) itself did not generate gp140-specific binding antibody responses.

**Figure 4:**
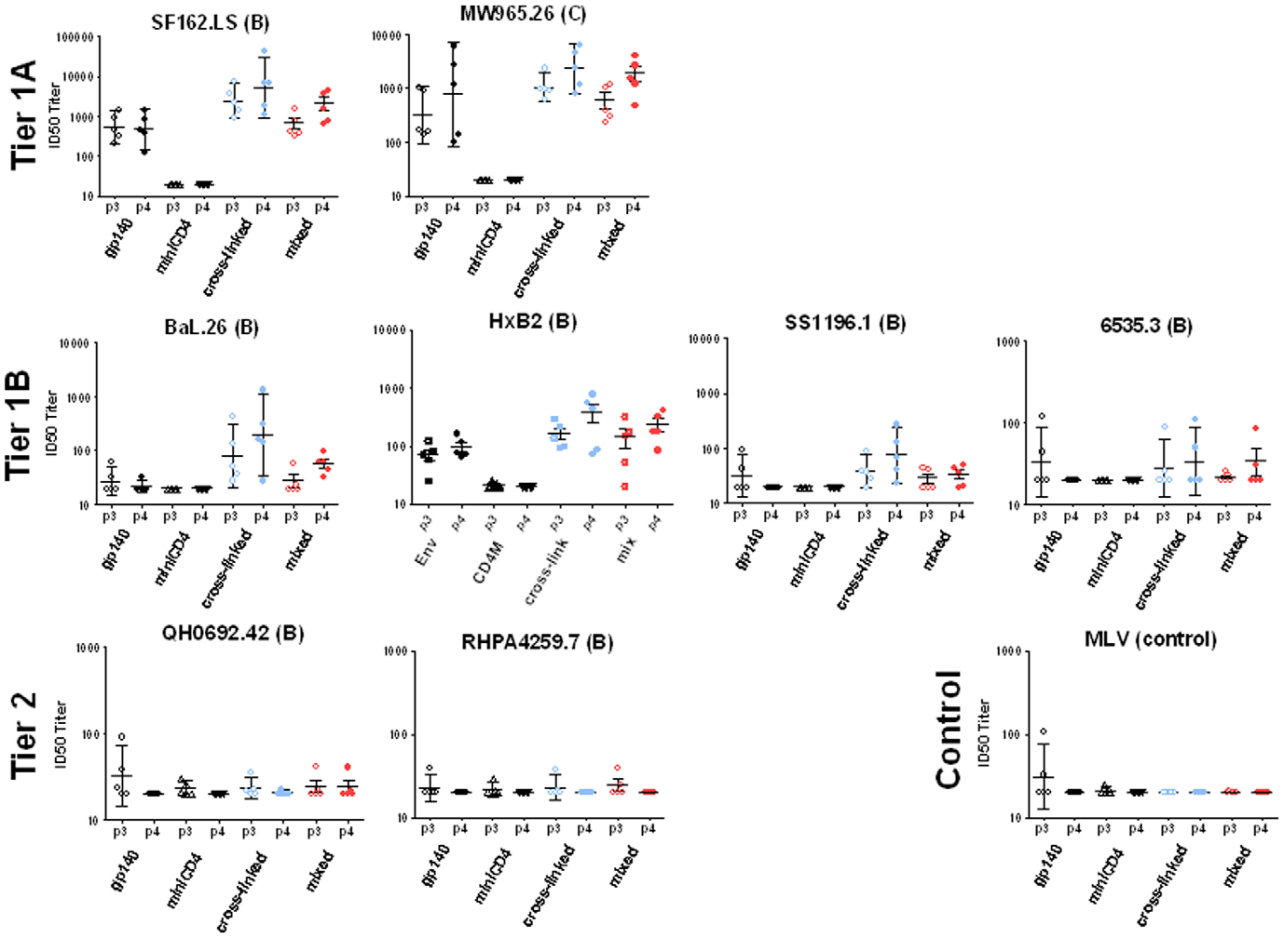
Neutralization of a panel of tiered HIV-1 Env pseudoviruses by vaccine sera. Analysis of breadth by neutralization of various tiered HIV-1 Env pseudoviruses by 2wp3 (p3) and 2wp4 (p4) sera from animals immunized with gp140 (open black circles, filled black circles), miniCD4 (M64U1) (open black triangles, filled black triangles) and two forms of the gp140-miniCD4 complexes: cross-linked (gp140-S-S-M64U1) (open blue circles, filled blue circles) and mixed (gp140+M64U1) (open red circles, filled red circles). ID50 neutralization titers are indicated against different viral isolates: SF162.LS, HxB2, MW965.26, BaL.26, SS1196.1, 6535.3, QH0692.42 and RHPA4259.7. The tiered categorization of the HIV-1 Env pseudoviruses is indicated. The subtypes are indicated within parenthesis. Neutralization of MLV is shown here as a control.

The avidity of gp140-specific serum antibodies was also measured using an ammonium thiocyanate (NH_4_SCN) ELISA (Fig. 3C) to estimate the extent of antibody maturation. Antibody avidity was similar two weeks following third (2wp3) or fourth (2wp4) immunizations (P>0.05 all groups except the M64U1 (miniCD4)-immunized group), although subtle increases were observed for both the gp140-miniCD4 [gp140-S-S-M64U1 (cross-linked) and gp140+M64U1 (mixed)] complex regimens between these two time points. The slightly higher avidities could potentially account for the improved virus neutralization seen with 2wp3/2wp4 sera from the gp140-miniCD4 (cross-linked or mixed) complexes (see below; Fig. 4). Four immunizations, with gp140 or the gp140-miniCD4 (cross-linked or mixed) complexes, elicited serum antibodies with the highest avidity (Fig. 3C).

### Immunization of rabbits with cross-linked gp140-miniCD4 (gp140-S-S-M64U1) complex elicited higher titer and broader HIV-1 neutralizing antibody responses than immunization with gp140 alone

Sera collected two weeks following the third (p3) and fourth (p4) immunizations were analyzed for their capacity to neutralize the homologous SF162 strain and a select panel of tiered heterologous HIV-1 Env pseudoviruses (Fig. 4). Sera were examined using the well-standardized pseudovirus-based TZM-bl reporter assay, as described in materials and methods. Sera obtained from rabbits immunized with the miniCD4 (M64U1) alone did not display neutralizing activity against any of the viruses at the maximum concentration tested (dilution of 1∶25) (Fig. 4). However, all remaining groups showed SF162 neutralization titers of >25, at both time-points tested (Fig. 3). Neutralizing titers increased following subsequent immunizations (p4>p3) with the gp140-S-S-M64U1 (cross-linked) or gp140+M64U1 (mixed) complex, but not with gp140 where the titers remained unchanged following fourth protein administration. Interestingly, groups immunized with the cross-linked or mixed gp140-miniCD4 complexes elicited higher SF162 neutralizing titers than groups immunized with gp140 alone, more so using the cross-linked complex (for 2wp4 sera, gp140 vs. both groups of complexes, p = 0.0127; gp140 vs. cross-linked group only, p = 0.0159. Fig. 4). This was also similar with regard to the neutralization of other Tier 1A virus, Subtype B HxB2 and Subtype C MW965.26, where the fourth immunization elicited higher neutralizing titers in all groups (except the control group, miniCD4). When the sera were evaluated for neutralization of Tier 1B viruses (Subtype B BaL.26 and SS1196.1), animals immunized with the cross-linked complex generated the highest titers of neutralizing antibodies following fourth immunization. When Tier 2 viruses (Subtype B 6535.3, QH0692.42, RHPA4259.7) were tested, the titers of neutralizing antibodies generated were very low or not measurable. Except for neutralization of 6535.3 pseudoviruses by sera of animals immunized with the gp140-miniCD4 complex (both cross-linked and mixed), no other sera showed any neutralization activity against Tier 2 viruses. None of the sera neutralized QH0692.42 or RHPA4259.7 pseudoviruses.

As expected, most of the immune sera tested did not neutralize MLV, here used as a control, except for low, non-specific neutralization by 2wp3 gp140-immunized sera (Fig. 4). Due to this observation, we negated the minimal neutralization of 6535.3 and QH0692.42 viruses by the same (gp140-immunized) sera.

When comparing the neutralization of Tier 1A (SF162, HxB2, and MW965.26) pseudoviruses, the IC50 values of 2wp3/2wp4 sera from the cross-linked complex were significantly higher for SF162 as compared to either of the mixed complex or gp140 alone [cross-linked vs. mixed: p3 = 0.0317 & p4 = 0.1508; cross-linked vs. gp140: p3 = 0.0159 (Fig. 4)]. HxB2 and MW965.26 neutralization titers using 2wp3 and 2wp4 sera showed no significant differences between groups. HxB2, despite being a lab-adapted Tier 1A virus, is not highly sensitive to anti-V3 antibodies. The HxB2 V3 loop contains a rare dipeptide insert (Gln-Arg) immediately adjacent to the Gly-Pro-Gly-Arg (GPGR) sequence at the V3 crown, a region highly targeted by antibodies generated during natural infection and upon vaccination by Env proteins [42]. Therefore, the improved neutralization of HxB2 virus by the sera from the cross-linked complex group (Fig. 4) indicated that the neutralization observed here was not primarily due to anti-V3 antibodies. To address this further, ‘epitope mapping’ experiments were performed and are described in section below.

The 2wp3/2wp4 sera from the cross-linked complex also neutralized the Tier 1B viruses consistently better than the sera from mixed complex or from gp140 alone. The IC50 values, for the Tier 1B neutralization by sera from cross-linked complex, were >1–2 log lower when compared to that from Tier 1A neutralization (Fig. 4). In case of Tier 2 viruses, only sera from the mixed complex (both 2wp3 and 2wp4) gave consistent, although low, neutralization of 6535.3 virus; the other Tier 2 viruses were not consistently neutralized by any sera (Fig. 4). Overall, it was evident that the 2wp3/2wp4 sera from the rabbits immunized with the cross-linked complex was most potent in neutralizing the various tiered HIV-1 isolates.

Overall, immunization with the cross-linked gp140-miniCD4 (gp140-S-S-M64U1) complex resulted in an increase in the breadth of HIV neutralization as compare to immunization with uncomplexed gp140 protein. Sera from animals immunized with the cross-linked complex neutralized the majority of pseudoviruses tested in the panel with neutralizing titers higher than sera from other groups. The sera from rabbits immunized with the mixed (gp140+M64U1) complex also neutralized >50% of the heterologous viruses, including one Tier1B and Tier 2 virus, albeit with low potency. In contrast, sera immunized with gp140 only neutralized the Tier 1A, SF162 and MW965.26, viruses.

### The cross-linked gp140-miniCD4 (gp140-S-S-M64U1) complex elicited binding antibodies directed against CD4i epitope(s) on HIV-1 Env

2wp4 sera were evaluated for specificities of antibodies elicited, using an ‘epitope mapping’ approach described previously [22]. Various forms of monomeric gp120, wild-type and mutants, were purified to >95% purity using method described previously [33]. Amongst the mutant gp120s, we generated the following: gp120D368R, a CD4BS mutant [43], [44], [45]; gp120I420R, a CD4i site mutant [46], [47], gp120ΔV3 (V3 deleted) and gp120ΔV1V2 (V1V2 deleted). The ability of the mutant gp120s, in comparison to wild-type, to exclude binding to known MAb was screened using SPR (data not shown). Here, we used both gp120I420R (CD4i site mutant) and cross-linked gp140-S-S-M64U1 complex (exposing CD4i epitopes) with the hypothesis that the two reagents could provide complementary information to validate the specificity of antibodies generated against the CD4i epitope(s). All gp120s and cross-linked gp140-S-S-M64U1 complex were then covalently coupled to Dynabeads (as described in Materials and Methods). Following coupling, the bead-coupled antigens were tested to determine their specificities to bind or abrogate recognition of well-characterized MAbs. For example, gp120ΔV3-coupled beads bound MAb 2G12, MAb b12, CD4-IgG2 and MAb 17b (in presence of sCD4), but not anti-V3 MAbs, 3109a and 2257; gp120I420R-coupled beads bound all above MAbs except 17b, in presence of sCD4; gp120D368R-coupled beads bound all above MAbs except CD4-IgG2 and MAb b12 (data not shown).

Rabbit sera from various groups were then subjected to differential solid-phase adsorption. From the gp140-immunized group, 2wp4-sera were selected from two animals showing the highest neutralization titers. From the group immunized with the cross-linked gp140-S-S-M64U1 complex, 2wp4-sera from all five rabbits were selected, whereas 2wp4-sera from only two animals were selected from the groups immunized with gp140+M64U1 (mixed) complex and gp140 alone. Sera from rabbits immunized with M64U1 (miniCD4) alone were not analyzed due to their lack of binding and neutralizing potency.

**Figure 5:**
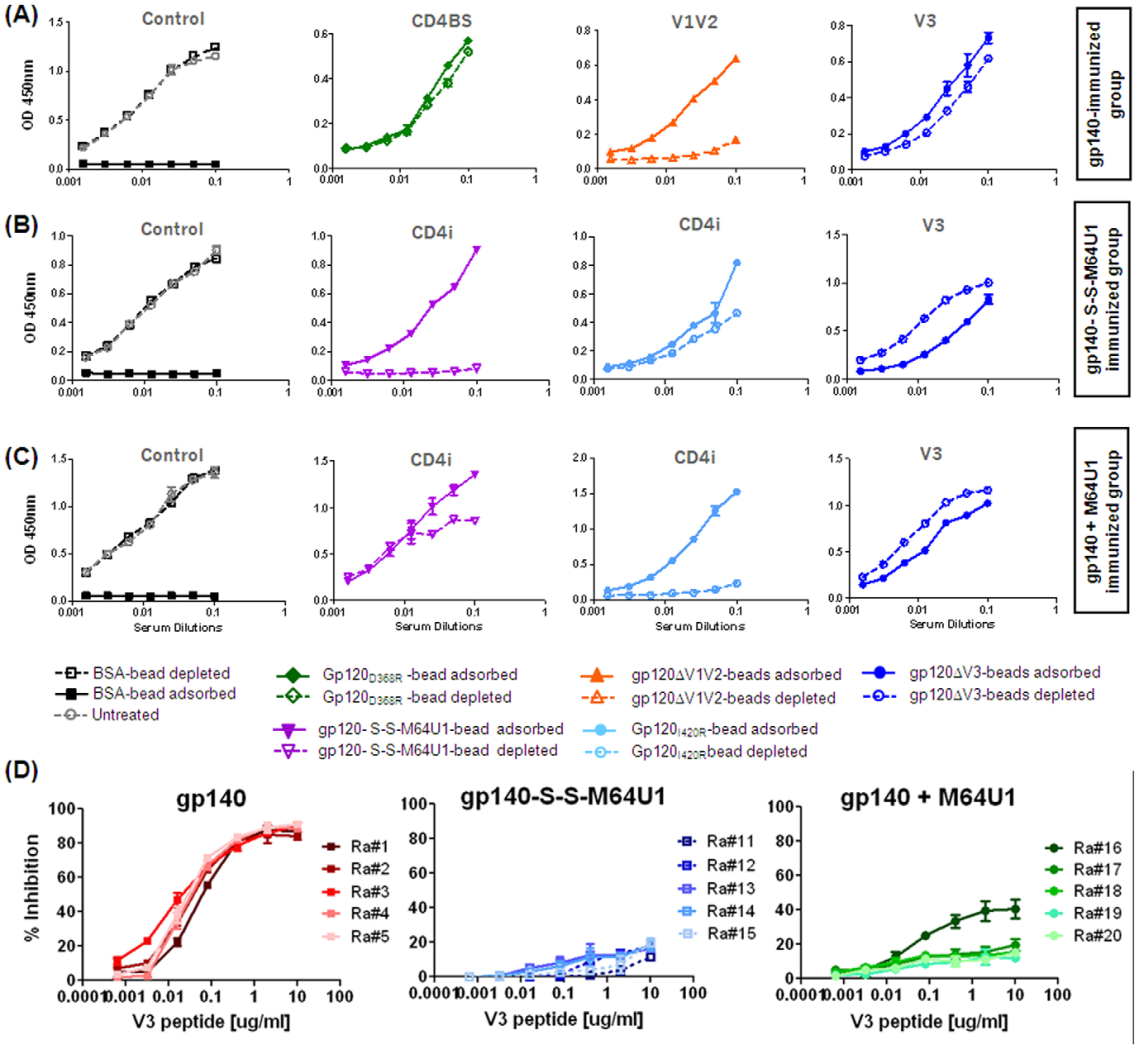
Serum-mapping analysis. Determining epitope-specificity of sera using (A–C) a differential solid-phase adsorption method [38], and (D) V3-cyclic peptide competition assay. A representative profile of the antibody-specificity generated by sera from each of the groups is shown upon adsorption by the various antigen-coupled beads, as described in Material and Methods. Specificity of sera from group immunized with (A) gp140, (B) cross-linked gp140-S-S-M64U1 complex and (C) mixed gp140+M64U1 complex. The epitopes are highlighted atop each panel in grey. The beads (BSA-beads; black/grey curves) used as ‘control’ are shown on the first column. The second column shows specificity to ‘CD4BS’-eptiope (using gp120_D368R_-beads; green curves) for gp140 immunized group and ‘CD4i’ epitope (using gp140-S-S-M64U1 complex; purple curves) for cross-linked or mixed gp140-miniCD4 complex immunized groups; the third column shows specificity for ‘V1V2’-loop (using gp120ΔV1V2-beads; orange curves) for gp140 immunized group and ‘CD4i’ epitope (using gp120_I420R_-beads; cyan curves) for cross-linked or mixed gp140-miniCD4 complex immunized groups. The final column shows specificity to ‘V3’-loop (using gp120ΔV3-beads; blue curves). (D) Competition assay to inhibit binding of gp140 (left panel) and gp140-miniCD4 complex, cross-linked gp140-S-S-M64U1 (middle panel) or mixed gp140+M64U1 (right panel), immunized rabbit sera to gp140 by V3-cyclic peptide. As control, a V3-scrambled cyclic peptide was used (see Materials & Methods for details). The percent (%) inhibition, which is the extent of serum binding to gp140 inhibited by V3-peptide, is calculated as the ratio of serum binding in presence of V3-cyclic peptide to serum binding in presence of control peptide ×100.

Upon adsorption, sera from animals immunized with gp140 generated antibodies predominantly directed to the gp120 subunit, evident by near-complete depletion of binding antibodies by gp120ΔV1V2-coupled beads (Fig. 5A). A proportion of these antibodies was V3-specific, and therefore could not be depleted by gp120ΔV3 proteins. Interestingly, sera from gp140-immunized animals also elicited substantial levels of anti-CD4BS antibodies. Post-adsorption of the sera with beads containing gp120D368R, a CD4BS mutant, a noteworthy level of antibodies remained in the depleted fraction, primarily due to their inability to bind to CD4BS (Fig. 5A). The depleted sera, but not the adsorbed fraction, were found to partially compete with CD4-IgG2 and CD4BS-MAbs when analyzed using SPR (data not shown), confirming their CD4BS-specificty. These sera from gp140-immunized rabbits, when treated with cross-linked gp140-S-S-M64U1 complex-coupled beads, could not be fully depleted of gp120-specific antibodies, highlighting that the depleted fraction still contained levels of non-CD4i antibodies (Fig. 5A). This also indicates that specific elicitation of CD4i antibodies was not feasible using gp140 alone.

**Figure 6:**
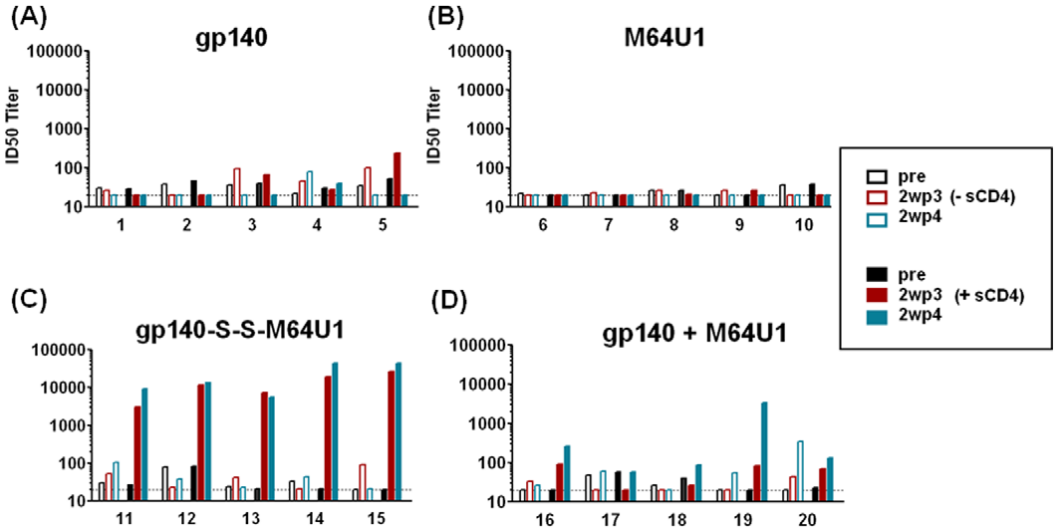
Neutralization of HIV-2 in the presence and absence of sCD4. Individual rabbit sera from the four groups (immunized with either gp140, miniCD4 M64U1, cross-linked gp140-S-S-M64U1 complex or mixed gp140+M64U1 complex) were tested for neutralizing activity against the isolate 7312A/V434A in the absence (open bars) or presence (filled bars) of sCD4. The ID50 neutralization titers are shown for the pre-immune (black), 2wp3 (red), and 2wp4 (blue) serum samples. The antigens used for immunization are indicated above each graph. Dashed lines indicate the lowest dilution tested.

The sera from animals immunized with gp140+M64U1 (mixed) complex and gp140 did not elicit substantial levels of CD4i antibodies. This was evident by comparing the adsorption of sera by both the gp120I420R-coupled and cross-linked gp140-S-S-M64U1 complex-coupled beads (Fig. 5C). All the binding antibodies in the sera were completely adsorbed by gp120I420R, indicating possible lack of antibodies to CD4i-site, whereas the depleted fraction showed no binding antibodies. On the other hand, upon adsorption using the complex-coupled-beads, binding antibodies were detected in both the adsorbed and depleted fractions (Fig. 5C). Taken together, these results indicated that this immunogen elicited low levels of CD4i-antibodies (also see Fig. 6). Some of the Abs in the sera was directed to the V3-region (Fig. 5C) and no CD4BS-antibodies were induced (data not shown).

When sera from the animals immunized with cross-linked gp140-S-S-M64U1 complex were analyzed, the predominant response was against CD4i epitope(s), and to some extent to the V3 loop (Fig. 5B). Interestingly, gp120I420R and gp140-S-S-M64U1complex-coupled beads generated complementary data, confirming the elicitation of CD4i antibody response in this group of rabbits (Fig. 5B). Gp120I420R-coupled beads failed to completely deplete gp120-reactive antibodies (Fig. 5B), whereas cross-linked gp140-S-S-M64U1 complex-coupled beads managed to do so. In the case of gp120I420R-coupled beads, both the adsorbed and depleted fractions showed significant amounts of gp120-specific antibodies, with CD4i antibodies dominating the depleted fraction. In sera treated with cross-linked gp140-S-S-M64U1 complex-coupled beads, all the gp120-specific antibodies fractionated in the adsorbed fraction, affirming the pull-down of antibodies to other gp120-epitopes including CD4i site which is readily exposed in this complex (Fig. 5B).

Bovine Serum Albumin (BSA)-coupled beads were used as control to show the specificity of adsorption. None of the sera displayed any non-specific adsorption to BSA (left panel, Fig. 5A, B, C).

Finally, to evaluate the relative levels of anti-V3 antibodies elicited by the three immunogens - gp140, gp140-S-S-M64U1 (cross-linked) and gp140+M64U1 (mixed), a V3-peptide competition assay was performed. Results showed that >80% of the binding activity to SF162 gp140 from the gp140-immunized sera could be inhibited by V3-cyclic peptide (left panel, Fig. 5D). Whereas, under similar conditions, ≤20% of the binding to gp140 from the two gp140-miniCD4 complex-immunized groups [gp140-S-S-M64U1 (cross-linked) and gp140+M64U1 (mixed)] (middle and right panel, respectively, Fig. 5D) was inhibited by the V3-cyclic peptide, except for sera from one animal (Ra#16, gp140+M64U1immunized group) which showed ≥40% inhibition at the highest V3-peptide concentration tested (right panel, Fig. 5D). This indicated that elicitation of anti-V3 antibodies was more pronounced following immunization with the gp140 immunogen as compared to the gp140-miniCD4 [gp140-S-S-M64U1 (cross-linked) and gp140+M64U1 (mixed)] complex immunogens.

### The cross-linked gp140-miniCD4 (gp140-S-S-M64U1) complex elicited high levels of antibodies directed against CD4i site that neutralized HIV-2_7312A/V434M_ in the presence of sCD4

Sera were next analyzed for the presence of virus neutralizing antibodies directed against the highly conserved CD4i site by testing their capacity to neutralize HIV-2 in the presence and absence of soluble CD4 (sCD4) [10]. Individual rabbit sera were tested from the pre-immune, 2wp3, and 2wp4 time points and the ID50 neutralizing titers determined (Fig. 6). In the gp140 group, serum from only a single rabbit (rabbit numbered 5) displayed ID50 titers of ≥2-log against the HIV-2_7312A/V434M_ pseudovirus in presence of sCD4, at 2wp3. However, at 2wp4, this CD4i neutralization was lost (Fig. 6A). As expected, none of the sera from the M64U1 (miniCD4)-immunized (control) group neutralized HIV-2 (Fig. 6B). Importantly, all animals immunized with the cross-linked gp140-S-S-M64U1 complex elicited highest neutralizing titers at 2wp3, with only modest improvement at 2wp4. 5/5 animals in this group neutralized HIV-2_7312A/V434M_ pseudovirus in presence of sCD4 with ID50 tiers of ≥4-logs, with rabbits numbered 14 and 15 showing the higher virus neutralization activities (Fig. 6C), confirming elicitation of strong CD4i antibody responses. In contrast, the rabbits that received the gp140+M64U1 (mixed) complex did not elicit comparable levels of CD4i antibodies, with only one rabbit (numbered 19) at 2wp4 that displayed an ID50 titer of 3–4 log (Fig. 6D).

These results indicated that generation of a stable complex of gp140-miniCD4 such as the cross-linked gp140-S-S-M64U1, rather than short-term mixing of the two proteins, was the best practical approach to elicit highest levels of CD4i neutralizing antibodies in this animal model.

## Discussion

Recent studies evaluating the evolution and specificities of broadly-neutralizing antibodies during HIV-1 infection [22], [23], [48] have provided important insights regarding the significance of CD4i antibodies and their potential role in vaccine against HIV-1. So far, recombinant monomeric gp120 or oligomeric/trimeric gp140 glycoproteins have failed to elicit broad and potent neutralizing antibodies in experimental animal models. Therefore, rationale structural alterations to soluble Env for effective presentation of conserved ‘neutralizing’ epitopes represent an important strategy towards HIV-1 vaccine development. Described herein is a practical approach of eliciting CD4i epitope-directed virus neutralizing antibodies using a stably cross-linked complex of recombinant oligomeric gp140 and miniCD4 (M64U1-SH) [27], [28], to target the conserved co-receptor binding site of the HIV-1 Env.

In earlier studies, two CD4 mimetic miniproteins (miniCD4) were cross-linked to various forms of HIV-1 Env [27], [28]. Based on results from those studies, the M64U1-SH miniCD4 was selected here for generating the cross-linked gp140-miniCD4 (gp140-S-S-M64U1) complex. Importantly, we observed that formation of a stable covalent complex by cross-linking of miniCD4 and gp140 protein via disulfide bond formation [27] resulted in significantly higher-titers of CD4i epitope-directed binding and virus neutralizing antibodies in rabbits immunized with the cross-linked (gp140-S-S-M64U1) complex, not seen in animals that received a ‘bench-side’ mixing of miniCD4 and gp140 (gp140+M64U1). Nevertheless, both miniCD4 groups [cross-linked or mixed] elicited antibodies of higher avidities (Fig. 3B) than the gp140 alone group, potentially contributing to the improved virus neutralization seen with 2wp3/2wp4 sera from the (cross-linked or mixed) complexes (Fig. 4).

Moreover, rabbits immunized with the cross-linked gp140-S-S-M64U1 complex elicited antibodies targeted to the CD4i epitope(s) while those immunized with gp140 alone or mixed (gp140+M64U1) complex did not. By ‘serum mapping’ experiments, it appeared that the majority of the animals (except the control group immunized with the miniCD4) elicited V3-specific antibodies. Only one animal in the gp140-immunized group elicited CD4BS antibodies (Fig. 5A) and all animals (5/5) immunized with cross-linked gp140-S-S-M64U1 complex elicited CD4i epitope-directed antibodies, whereas the mixed (gp140+M64U1) complex did not. This observation of selective elicitation of CD4i epitope-directed antibodies was confirmed by the HIV-2 neutralization assay (Fig. 6).

Subtype B immunogens, such as the SF162 gp140 used here, elicit substantial levels of anti-V3 antibodies that can be blocked by subtype-specific V3 peptide [49]. In addition, Env undergoes a conformational change upon CD4 binding that results in the relocation of the inner domain of gp120 to the molecular surface and exposure of V3 loop from the core domain to the solvent-accessible surface of gp120 [50], [51]. Therefore, the induction of anti-V3 antibody responses by the gp140-miniCD4 [gp140-S-S-M64U1 (cross-linked) and gp140+M64U1 (mixed)] complex immunogens was evaluated using a competition assay that employed V3-cyclic peptide (Fig. 5D). While over 80% of the gp140-specific binding activity of sera from gp140-immunized group was inhibited by V3-peptide, only about 20% of the binding activity of sera from both gp140-miniCD4, cross-linked and mixed, complex immunogen immunized animals was inhibited by V3-peptide. This observation that the elicitation of anti-V3 antibodies was not enhanced by both gp140-miniCD4 complex immunogens was also supported by HxB2-neutralization results. Sera from both the gp140-miniCD4, cross-linked or mixed, complex immunized groups neutralized HxB2, a virus that is not sensitive to anti-V3 antibodies [49], more potently (although without statistical significance) than sera from the group that was immunized by gp140 alone (Fig. 4). These data suggested that the structural alterations upon Env-CD4 protein binding and alterations upon gp140-miniCD4 interaction may differ concerning V3 loop exposure and subsequent V3-specific antibody elicitation. In addition, the results here also confirm that the breadth and potency by sera from cross-linked (gp140-S-S-M64U1) complex group is likely due to the elicitation of antibodies directed to CD4i epitope(s) rather than to the V3 loop. Hence, the use of a stable, covalently cross-linked complex of gp140 and miniCD4 appears to represent a viable method for the elicitation of higher titer antibodies directed to conserved CD4i epitope(s) on HIV-1 Env.

It was reported previously that some CD4i full-length antibodies encounter steric hindrances in accessing the ‘induced’ epitope on viral envelope glycoprotein available post-CD4 attachment, due to constrained space between the cell and the viral membrane following (viral) Env and (cellular) CD4 interaction [17]. Nevertheless, numerous studies have reported the elicitation of high titers of CD4i antibody during natural human infection against multiple HIV-1 subtypes and circulating recombinant forms (CRFs). Importantly, these antibodies bind and neutralize not only various HIV-1 isolates but also HIV-2 in CD4-induced manner [23], indicating the functional constraints on receptor binding that create opportunities for broad humoral immune recognition and neutralization of viral quasispecies. Although speculations could be made as to how these antibodies are so commonly generated and how they neutralize various HIV-1 isolates, the precise *in vivo* mechanism, more importantly in neutralization, is unknown.

The elicitation of CD4i antibodies signifies the extraordinary degree of antigenic conservation linked to co-receptor binding exhibited by diverse HIV-1 and HIV-2 lineages, and at the same time, the ability of human humoral immune system to exploit these constraints [23]. But since humoral response determined by analyzing circulating antibodies are likely to change significantly over time, due to the high mutability of HIV-1 envelope, this measure in sera is unlikely a reflection of long-term protection. Rather analyses of memory B-cell compartment may be more telling about protective immunity. Recently, Y Guan et al. reported that although “elite controllers” showed low level of circulating antibody response against CD4i epitope(s), CD4i epitope-specific B lymphocyte memory cells were present at high frequencies (greater than a third of the anti-Env antibodies) in all ‘elite controllers’ subjects tested [52]. Besides, similar conclusion on broader diversity of anti-HIV-1 antibodies isolated from memory B-cells have been echoed by JF Scheid et al. [53], in which ≥15% of the neutralizing antibodies were directed to the CD4i epitope(s). The induction of CD4i Abs in patients infected by HIV-1 [22], [23], [52] highlights the exposure of the co-receptor binding site on the virus surface, which may occur after binding of gp120 to CD4 on the target cell despite the steric hindrance reported by Labrijn et al. [17], or because of CD4-independent variants exposing the co-receptor binding site in most of the infected patients [10], [54]. Presence of circulating soluble CD4 or of CD4-gp120 complexes at the surface of targeted cells, following shedding of gp120 from gp41 and consequently from the virus particle following HIV-1 binding to CD4, could also contribute to elicitation of CD4i antibodies, as most recently exemplified in primates [55]. Overall, these and other studies highlight the role of CD4i antibodies during the course of HIV-1 infection.

In the past, several studies have been performed, based on gp120-CD4 (or CD4 mimic) complexes or constrained ‘core’ gp120 antigens as vaccine candidates, aiming at inducing CD4i antibodies [18], [19], [56], [57], [58]. T Fouts et al. showed that gp120 cross-linked to CD4 D1D2 domains raised antibodies that neutralized primary viruses regardless of co-receptor usage and genetic subtype in nonhuman primates [18]. These findings were extended in a challenge study by A DeVico et al. [19], where macaques immunized with a single chain complex containing gp120BaL-rhesus macaque CD4 D1D2 showed better CD4i antibody response that correlated with the control of infection when challenged with SHIV162P3. Although this correlation is not a proof of the neutralizing efficiency of CD4i antibodies, it shows that the presence of these MAbs is fully related to the CD4-bound conformation of HIV-1 envelope *in vivo*. Besides, recent approach towards constraining ‘gp120 core’ protein by site-directed disulfide linkage(s) has been a novel step towards eliciting epitope-specific response against the co-receptor binding site [58]. These vaccination studies indicate the potential importance of strategies directed to raising antibodies against the CD4i site.

Some success in protection against HIV-1 acquisition has been afforded by active prophylactic vaccination using either DNA prime-recombinant Env protein boost [59], [60] or viral vector prime-recombinant Env protein boost [61], [62], [63], [64], [65], [66] or adjuvanted recombinant Env proteins [67], [68] in studies performed in nonhuman primate models, and most recently, in humans using a Canarypox vector prime plus recombinant Env protein boost approach in the RV144 Phase III trial performed in Thailand [69]. Recent correlates of risk assessments of the RV144 trial indicated that V1V2-directed IgG antibodies in sera from vaccinees were associated with vaccine-elicited protection [70]. Despite this early “hypothesis generating” finding in this first promising HIV-1 vaccine trial, the importance of the diversity of antibodies elicited by other epitopes on Env such as the CD4i epitope(s) [71], during infection and vaccination, and their role in immune protection against HIV-1 infection warrant further investigation.

The present work provides a powerful next generation approach using a novel and stable cross-linked complex of oligomeric gp140 glycoprotein and a CD4 mimetic miniprotein, in order to elicit broadly reactive functional antibody responses targeted to the highly conserved co-receptor binding site of the HIV-1 Env. Moreover, it does so while avoiding the elicitation of undesirable anti-CD4 reactivity. This cross-linked gp140-miniCD4 (gp140-S-S-M64U1) complex is suitably stable for future vaccine studies in non-human primate models, and represents a viable strategy for further evaluations of the role of CD4i epitope-directed antibody responses in protection against HIV-1 infection during vaccination.
